# Supplementary material for: Expression of a CO2-permeable aquaporin enhances mesophyll conductance in the C4 species Setaria viridis
Source: eLife. 2021 Nov 29;10:e70095. doi: 10.7554/eLife.70095 (PMC8648302; doi:10.7554/eLife.70095)
Supplement: Figure 2—source data 1. [file elife-70095-fig2-data1.docx]

**Figure 2 - Source data 1. Uncropped images of western blots**

Figure 2-source data 2


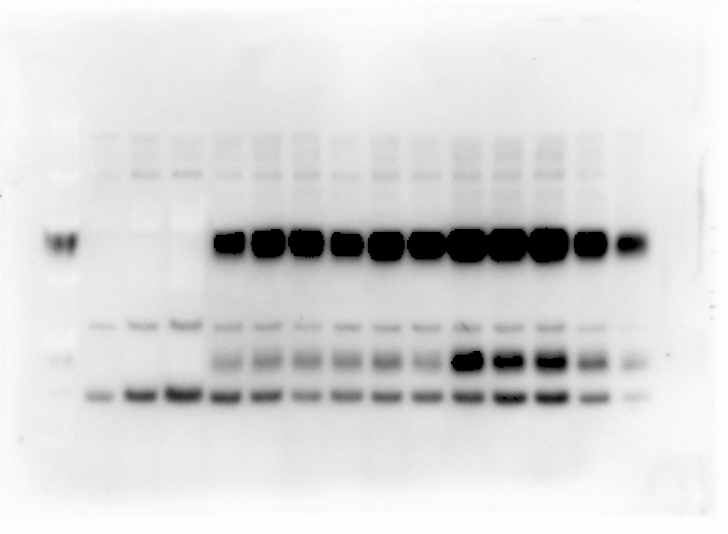


SiPIP2;7-FLAG

Rieske

Figure 2-source data 3


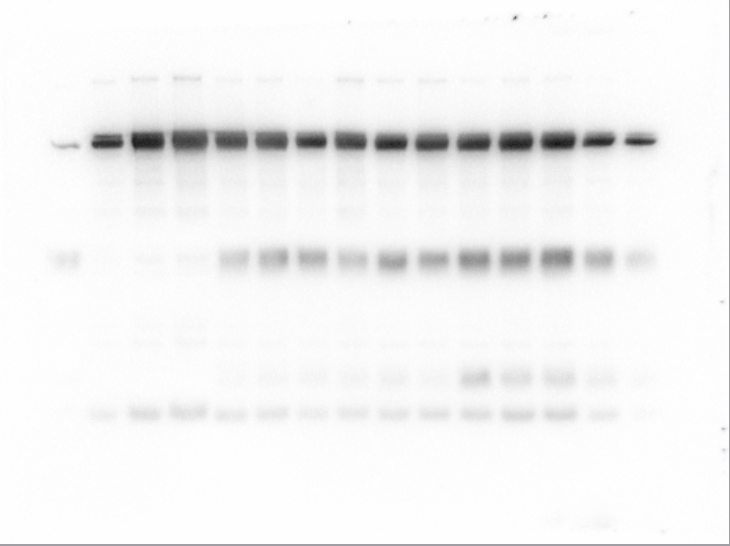


PEPC

Figure 2-source data 4


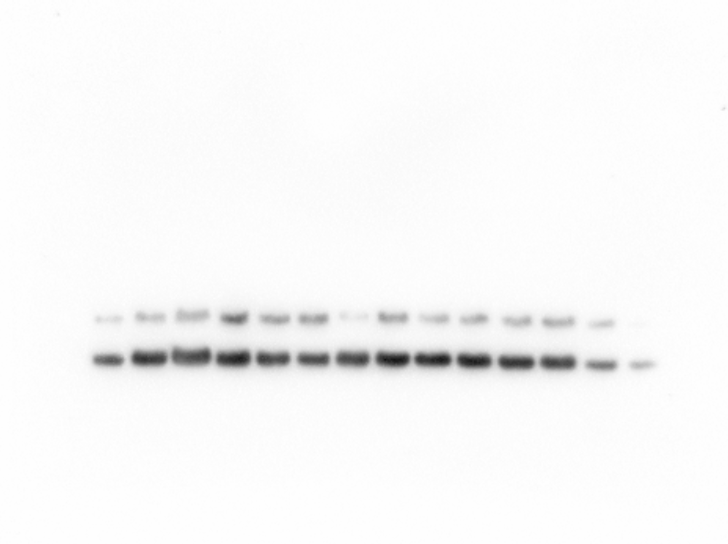


CA

Figure 2-source data 5


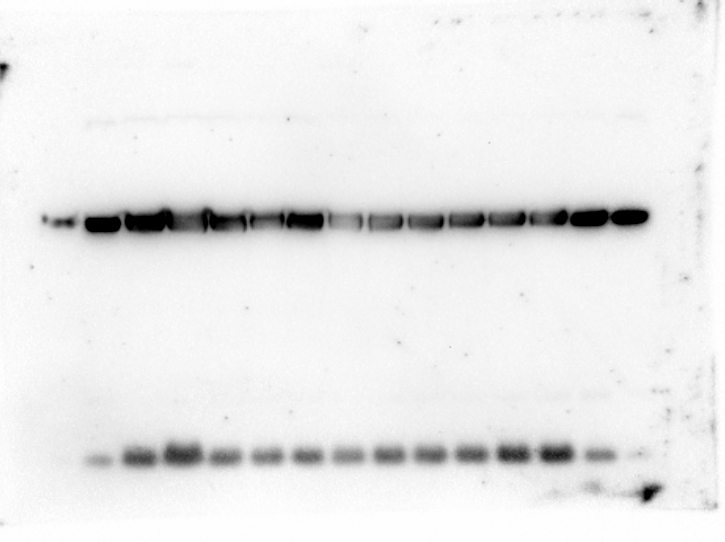


RbcS
